# Hyperarousal, Dissociation, Emotion Dysregulation and Re-Experiencing—Towards Understanding Molecular Aspects of PTSD Symptoms

**DOI:** 10.3390/ijms26115216

**Published:** 2025-05-29

**Authors:** Aleksandra Brzozowska, Jakub Grabowski

**Affiliations:** 1 Adult Psychiatry Scientific Circle, Division of Developmental Psychiatry, Psychotic and Geriatric Disorders, Department of Psychiatry, Faculty of Medicine, Medical University of Gdansk, 80-282 Gdansk, Poland; abrzozowska@gumed.edu.pl; 2 Division of Developmental Psychiatry, Psychotic and Geriatric Disorders, Department of Psychiatry, Faculty of Medicine, Medical University of Gdansk, 80-282 Gdansk, Poland

**Keywords:** stress-related disorders, post-traumatic stress disorder, complex post-traumatic stress disorder, molecular mechanisms of PTSD

## Abstract

Approximately 70% of people will experience a traumatic event in their lifetime, but post-traumatic stress disorder (PTSD) will only develop in 3.9% and complex post-traumatic stress disorder (CPTSD) in 1–8% of the population worldwide, although in some countries (e.g., Poland and Northern Ireland) it will develop in a much higher percentage. Stress-related disorders have a complex pathogenesis involving neurophysiological, genetic, epigenetic, neuroendocrine and environmental factors. This article reviews the current state of knowledge on the molecular aspects of selected PTSD symptoms: hypervigilance, re-experiencing, emotion dysregulation and dissociation, i.e., the symptoms with strong neurobiological components. Among analysed susceptibility factors are specific gene polymorphisms (e.g., *FKBP5*, *COMT*, *CHRNA5*, *CRHR1*, *5-HTTLPR*, *ADCY8* and *DRD2*) and their interactions with the environment, changes in the HPA axis, adrenergic hyperactivity and disturbances in the activity of selected anatomical structures (including the amygdala, prefrontal cortex, corpus callosum, anterior cingulate gyrus and hippocampus). It is worth noting that therapeutic methods with proven effectiveness in PTSD (TF-CBT and EMDR) have a substantial neurobiological rationale. Molecular aspects seem crucial when searching for effective screening/diagnostic methods and new potential therapeutic options.

## 1. Introduction

### 1.1. Complex Symptomatology of PTSD and CPTSD

Post-traumatic stress disorder (PTSD) develops as a consequence of exposure to one or more traumatic events, i.e., actual or threatened death, serious injury or sexual violence. PTSD can be diagnosed in a person who has experienced such an event personally, has been an eyewitness to it, has learned that a close family member or close friend has died or been exposed to death as a result of violence or an accident, or has experienced repeated or extreme exposure to details of traumatic events (e.g., firefighters, paramedics and policemen). Symptoms of PTSD vary in type and severity from patient to patient. They include recurrent, intrusive and involuntary memories, nightmares or flashbacks of the traumatic event, dissociative symptoms, avoidance of memories and trauma-related triggers, disturbances in emotional state (e.g., anxiety, anger, shame and lack of positive emotions) and cognitive functioning (e.g., negative beliefs about oneself or the world). Changes in excitability and reactivity are also characteristic and can include aggressive behaviour, risk-taking behaviour, hypervigilance and sleep disorders. The symptoms must last for more than a month [1].

Individuals exposed to chronic victimisation have a different clinical presentation than those with PTSD, hence the need to distinguish a new disorder [2,3,4]. Van der Kolk et al. developed the concept of Developmental Trauma Disorder (DTD), which is characterised by affect dysregulation (e.g., extremely negative affective states), dysregulation of attention and behaviour (e.g., self-harm), somatic dysregulation (e.g., hypersensitivity to touch or sounds), limited access to one’s own emotions and problems with expressing them (e.g., alexithymia), attachment disorganisation, distorted perception of oneself and the world, problems in interpersonal relationships and decreased functioning [2,5].

While the DSM-5 does not recognise DTD, the ICD-11 has included a new diagnosis—complex post-traumatic stress disorder (CPTSD)—which largely covers the DTD criteria but applies also to adults. CPTSD is a disorder that develops because of exposure to repetitive traumatic experiences that are difficult or impossible to escape (e.g., torture, slavery, genocide campaigns, prolonged domestic violence, repeated childhood sexual or physical abuse) [6]. Compared to PTSD symptomatology, CPTSD is additionally characterised by severe and persistent problems with affect regulation, distorted self-image and consistent emotionality (diminished, defeated and worthless), as well as problems with attachment and maintaining relationships [6,7]. Childhood cumulative trauma is a predictor of increased symptom complexity in adults with CPTSD [8]. There are many diagnostic instruments for PTSD and its specific domains. The gold standard for diagnosing PTSD is the Clinician-Administered PTSD Scale for DSM-5 (CAPS-5), which is a structured interview that takes into account all PTSD symptoms according to DSM-5 [9,10]. Another popular instrument is the PTSD Checklist for DSM-5 (PCL-5) that can be used for screening (e.g., in primary health care). It is a questionnaire examining 20 symptoms of PTSD according to the DSM-5 [11,12]. The Short Post-Traumatic Stress Disorder Rating Interview (SPRINT) consists of eight items relating to key symptoms of PTSD (intrusion, avoidance, arousal and numbing), impairment of daily functioning, coping with stress, somatic symptoms, as well as two items assessing dynamics of the disorder [13]. The International Trauma Questionnaire (ITQ) is a self-report tool consisting of 18 items that allows for the assessment of PTSD symptoms and disturbances of self-organisation, which together constitute CPTSD according to the International Classification of Diseases 11 [14,15]. To assess and monitor the severity of dissociative symptoms, the Dissociative Experiences Scale (DES-II) and the Multiscale Inventory of Dissociation (MID-60) are used [16,17,18]. In the context of emotion regulation disorders, the Posttraumatic Risky Behaviours Questionnaire (PRBQ), which examines involvement in risky or self-destructive activities, may be useful [19,20].

### 1.2. Complex Pathogenesis of Post-Traumatic Stress Disorders

Pathogenesis of post-traumatic stress is not fully explained, with the specific nature of this disorder (especially in the case of developmental trauma) sometimes making it difficult to distinguish the cause from the consequences of the trauma [21]. It is known that pathogenesis of PTSD includes abnormalities at the neuroanatomical [22,23,24], neuroendocrine [25,26,27], genetic [28,29,30,31] and epigenetic [23,29,32,33] levels, with these biological factors interacting significantly with the environment [34].

The heritable component of PTSD is polygenic, involving genes involved in the hypothalamic–pituitary–adrenal (HPA) axis, serotonergic, dopaminergic, noradrenergic, GABAergic, BDNF, NPY and APOE systems, among others [30,35,36,37]. Boscarino et al. indicated that the number of specific alleles of genes associated with PTSD risk (among *FKBP5*, *COMT*, *CHRNA5* and *CRHR1*) is related to the likelihood of PTSD during life, as well as to the age of onset of first symptoms. Individuals with more high-risk alleles and more exposure to trauma have an increased risk of PTSD and an earlier age of onset. Individuals with no or few high-risk alleles are resilient to PTSD, regardless of trauma exposure [36].

Furthermore, it is known that the risk of developing PTSD depends on gender, type of trauma, developmental period, personality factors, ethnicity, level of education, history of previous mental disorders (personal or in the family), cognitive abilities, coping and response styles and other biographical factors [38,39,40]. Also, previous exposure to traumatic events raises the risk of developing PTSD in the future [41].

### 1.3. Hyperarousal, Dissociation, Emotion Dysregulation and Re-Experiencing as Neurobiological Processes Typical for Post-Traumatic Stress Disorder

In this article, we want to focus on molecular aspects of four particular symptoms of PTSD, for which significant neurobiological correlates can be identified:

- **Hyperarousal**—defined as a high level of physiological arousal and excessive alertness to possible dangers or difficulties. In the course of PTSD, hyperarousal becomes dysfunctional because it automatically and uncontrollably connects with memories of traumatic events [42,43].
- **Dissociation**—loss of continuity of subjective experience due to unwanted intrusions associated with the traumatic memory, lack of access to information or control of mental functions, or the experience of detachment from oneself or reality [1,44]. When traumatic experiences are so difficult that they cannot be fully integrated, structural dissociation of the personality can occur [45,46,47].
- **Emotion dysregulation**—closely related to dissociation, especially in people with CPTSD [48]. This is a deficit of management of processes essential for emotional control, most likely related to neurofunctional disturbances associated with chronic traumatisation [48,49,50].
- **Re-experiencing**—a constant feature of stress-related disorders. It encompasses intrusive memories or images, flashbacks, repetitive dreams or nightmares thematically related to the traumatic event [18,51].

The above phenomena will be discussed in terms of pathogenesis, modulators and susceptibility to PTSD development.

## 2. Hyperarousal

During a traumatic event, being alert to alarm signals is a life-saving skill. Soldiers, for example, can increase their chances of survival if they recognise signals of an incoming air strike in time. Vigilance is even considered a part of a soldier’s ethos [52]. Individuals who have been subjected to chronic trauma are focused on searching for alarm signals because this can potentially protect them from harm (e.g., damage to health, injury or even death). This type of automatic processing of threatening stimuli is crucial for survival, but it persists even after the stimuli have ceased, playing a key role in the aetiology and maintenance of anxiety disorders [53]. Such attentional bias can develop not only when consciously processing threat but also with subliminal exposure to trauma-related stimuli. In the study by Rabellino et al. [54], PTSD individuals showed increased activation of the innate alarm system (i.e., the cerebellar–limbic–thalamo–cortical network), especially with subliminal stimuli. In hypervigilant individuals, visual scanning and arousal are higher not only when processing threatening stimuli, but also neutral ones, regardless of self-reported anxiety [52]. This can also be considered as executive function disorders with possible dysfunctions of the dorsal prefrontal networks [55] and irregularities in dopamine function [56]. Impairments in attention regulation and response inhibition are among the most robust deficits in PTSD. They are both a risk factor and an element of the clinical presentation and are related to the severity of symptoms [57]. Behavioural inhibition in childhood (i.e., restraint in engaging with the world and tendency to scrutinise the environment for potential threats) [1] may be associated with the development of attentional bias in adolescents [58].

Individuals with PTSD function as if the trauma was still ongoing, which is reflected also in their bodily reactions [59]. Traumatized individuals have altered stress response systems and acute stress reactivity compared to healthy individuals [60,61,62,63]. In the Trier Social Stress Test (TSST), individuals with PTSD had lower levels of cortisol and higher levels of salivary alpha-amylase (sAA), the latter of which is considered a reliable marker of autonomic nervous system activity [64]. There are neurofunctional connections here, as sAA reactivity is linked to the activation of the right amygdala (lasting even 20 min after the stressor) and the right dorsal anterior cingulate cortex [65]. Clear neuroimaging lateralization has also been demonstrated: in response to stress stimuli, the salience network is activated in the right rather than the left hemisphere, which confirms the hypotheses of asymmetry in stress reactivity [66], stress sensitivity and the dominance of the right hemisphere during the activation of traumatic memories [65]. The right hemisphere is responsible for assessing the emotional significance of incoming information and the subsequent regulation of hormonal and autonomic responses, while the left hemisphere is responsible for cognitive analysis [67] and exerts an inhibiting effect on the activity of the HPA axis [68]. When stress becomes chronic or is perceived as uncontrollable or impossible to escape, a shift from the initial dominant activity of the left medial prefrontal cortex (mPFC) to the right mPFC occurs, which activates a physiological stress response [68]. Also, numerous studies have shown that the size of the corpus callosum is significantly smaller in people with PTSD (especially in maltreated children with PTSD) [69,70,71]. As the corpus callosum is a structure connecting the two hemispheres, it is speculated that such abnormalities may impair processing of new information, especially traumatic events [71], and impair inhibitory callosal effects [72].

To understand the molecular differences that occur in people with hyperarousal-type PTSD, it is worth referring to LeDoux’s classical model of emotion processing. According to LeDoux, the amygdala plays a key role as a centre for assigning meaning to stimuli that arrive from the environment and are registered in the thalamus. Stimuli that are assessed as significant, e.g., those of an imminent threat, are processed via a preferential direct pathway (the so-called ‘low pathway’). The amygdala instantly sends signals to the hypothalamus and brainstem, which results in activation of the autonomic nervous system and secretion of cortisol and catecholamines—key drivers of stress reactions. Only then does a conscious interpretation of the stimuli take place, with the anterior cingulate cortex (ACC) and prefrontal cortex (PFC) playing a key role [73,74,75,76,77]. Considering the prominent role of the HPA axis and catecholamines in maintaining the state of hyperarousal, the molecular factors associated with them were analysed, with attention to potential susceptibility factors. Changes in glutamatergic transmission in the course of PTSD were also discussed as a key factor regulating the HPA axis response in the state of stress.

Table 1 summarizes the most important information about the molecular correlates of hyperarousal.

### 2.1. HPA Axis

PTSD is characterised by endocrine changes that are distinct from other mental disorders. These differences are particularly evident in the HPA axis. Cortisol levels are low in people with PTSD [26,78,79,80], which seems unintuitive because one would expect that symptoms such as re-experiencing would maintain the stress response in the chronic stage, stimulating hypercortisolism [78]. This phenomenon is explained by enhanced negative feedback inhibition. Following the HPA axis from the top, both depression and PTSD are associated with increased CRF secretion in the hypothalamus. In depression, according to the classical stress response pattern, this leads to increased ACTH secretion and subsequent hypercortisolism, which in turn inhibits the pituitary gland. In PTSD, however, despite increased CRF secretion, there is no increased cortisol secretion [25,26,81]. Hypoactive HPA axis leads to prolonged and elevated arousal to threat and hinders returning to baseline [82]. It is believed that hypocortisolism after a traumatic event is a predictor of PTSD development [83,84,85,86].

According to current knowledge, the most plausible explanation is that in traumatised individuals, the response to ACTH is suppressed at the level of the pituitary gland. It is assumed that the pituitary gland is hypersensitive to cortisol due to an increased number of glucocorticoid receptors (GR). Studies using the dexamethasone suppression test have confirmed that the key element responsible for the increased negative feedback axis are processes at the pituitary level [85,87]. One of the extensively studied genes is *FKBP5* encoding FK506 binding protein 5 which regulates glucocorticoid receptor sensitivity and is involved in the regulation of the HPA axis [36,88,89,90]. Binder et al. studied polymorphisms of this gene in the context of child-abuse trauma. Although SNPs of this gene did not directly predict the occurrence of symptoms in non-child abuse, four SNPs together with the severity of child abuse did predict the level of PTSD symptoms in adults. The association remained statistically significant when controlling for severity of depression, age, gender, level of exposure to non-child abuse and family factors [90]. The gene is worth looking at in a functional context. Its product binds to GR chaperone protein during maturation of the GR complex. Polymorphisms of this gene are responsible for the reduced sensitivity of GR to cortisol. In such a situation, the feedback loop in the HPA axis does not function efficiently, which potentiates the physiological stress response [90,91]. The chronic nature of the stress response is one of the key biological aspects of PTSD. The gene is subject to environmental influences, being an example of gene–environment (GxE) interaction in the pathogenesis of PTSD [36,92,93].

Further analysis of the HPA axis should also consider the polymorphisms of the corticotropin-releasing hormone receptor gene (*CRHR1* and *CRHR2*) [36,82]. In a study of child accident victims, a polymorphism of *CRHR1* was identified that increases susceptibility to acute PTSD symptoms and affects the course of symptoms in the future (rs12944712) [94]. The variants rs12938031 and rs4792887, on the other hand, were associated with the occurrence of PTSD in victims of the 2004 hurricane in Florida [95]. In the context of exposure to chronic trauma, the study by Sanabrais-Jimenez et al. [96] is particularly relevant, as they demonstrated the interaction of *CRHR1* and *CRHR2* with childhood trauma and an increased risk of suicide attempts. This particularly applies to the following types of trauma: physical neglect, emotional abuse and sexual abuse, which, in combination with certain genetic variants, increase the risk of suicide.

### 2.2. Glutamatergic and GABAergic Activity

The HPA stress responses are integrated by glutamatergic and GABAergic systems. The paraventricular nucleus (PVN) of the hypothalamus is densely innervated by glutamatergic and GABAergic neurons [97,98,99,100]. In a brain that is not subjected to severe stress, depression (or some other disorders, e.g., autism spectrum disorder, schizophrenia or substance abuse), there are effective regulatory mechanisms that maintain a balance between excitatory Glu and inhibitory GABA [101,102,103]. There are several pathways for the synthesis of GABA from Glu, one of which is glutamic acid decarboxylase (GAD). In individuals with PTSD, the balance between Glu and GABA is disrupted in favour of excitatory Glu. In an animal model, it has been shown that chronic adverse stimulation reduces the expression of one of the isoenzymes (GAD67) in the hippocampus, amygdala and PFC leading to diminished inhibitory impulses to the PVN [104]. Another reason for reduced GABAergic signalling to the PVN is glutamatergic damage to the hippocampus [104,105,106,107]. This leads to a reduction in PVN inhibition, loss of precise control over the HPA axis and disrupted termination of the stress response [104]. Hyperarousal is also caused by glutamate-induced pathological neuroplasticity in the amygdala and PFC [108,109]. In the amygdala, excitability increases, and the prefrontal cortex has weakened inhibition of the amygdala, which means that it does not effectively inhibit stress responses [110,111]. NMDA receptors in the amygdala promote neuronal changes that cause stress learning [112].

### 2.3. Catecholaminergic Activity

Alterations in the adrenergic system are also of great importance in the pathomechanisms of PTSD. Despite a lack of full consensus, it is assumed that people with PTSD have elevated basal levels of catecholamines [78,113]. An increased psychophysiological and hormonal response to trauma-related stimuli seems more important. As with the HPA axis, individuals with PTSD show a certain hypersensitivity in the adrenergic system. This may be due to a reduced number of alpha-2 autoreceptors, whose stimulation inhibits the secretion of noradrenaline [78]. The administration of a selective alpha-2 receptor antagonist (yohimbine) led to an increased secretion of noradrenaline and triggered PTSD symptoms [114]. With fewer alpha-2 receptors, the inhibition of noradrenaline secretion is reduced.

*COMT* is the gene encoding catechol-O-methyltransferase, an enzyme involved in the breakdown of catecholamines such as dopamine, epinephrine and norepinephrine [115,116]. These are neurotransmitters that play an important role in the stress response. In terms of the genetic basis of PTSD, a SNP within codon 158 (substitution of valine for methionine) resulting in a 3- or 4-fold reduction in enzyme activity appears to be relevant [117,118]. The *COMT* Val158Met polymorphism is associated with reduced resilience to stress, reduced ability to extinguish conditioned fear and the risk of developing PTSD after exposure to multiple traumatic experiences. Kolassa et al. showed that greater exposure to multiple traumatic events correlates with higher incidence of PTSD in a dose-dependent manner, but this relationship is modulated by the *COMT* polymorphism, with Met/Met homozygotes having the highest risk of developing the disorder [119]. The disturbed breakdown of catecholamines resulting from this polymorphism appears to be relevant in different types of trauma: urban violence [120], war trauma [121], natural disasters [122] and in different age groups [123]. Furthermore, *COMT* polymorphisms appear to be associated with hippocampal activation and memory impairment in people with PTSD, including those with early childhood trauma. Val/Val homozygotes (i.e., non-mutant) experience increased hippocampal activation in response to trauma, which correlates negatively with PTSD and depression and promotes resilience. In contrast, carriers of two Met alleles respond with reduced hippocampal activation [124,125]. One explanation for the greater susceptibility of Met/Met homozygotes to PTSD may be that the Val158Met polymorphism is associated with arousal in response to traumatic events. In contrast, individuals with Val/Val alleles respond with depressive symptoms [123]. Met/Met homozygotes have major problems in extinguishing the stress response [126].

### 2.4. Molecular Networks

Attempts are being made to explain the pathophysiology of PTSD using molecular networks. This approach addresses the complexity of its pathogenesis, with multiple interplays between genetic, neuroendocrine and other biological factors and environmental exposure. Neylan et al. argue that PTSD symptoms should be treated as the visible properties of complex molecular networks, as opposed to processes driven by a small number of genes [127]. This approach uses a wide range of data, including DNA, RNA, proteins, metabolites, clinical data, imaging data and available literature.

### 2.5. Dynamics of Changes After Childhood Trauma

When it comes to trauma experienced at a young age, neuroendocrine changes occur gradually, as proven by Pervanidou et al., who conducted a longitudinal study on children and teenagers after a motor accident [128]. They examined the cortisol and catecholamine levels in serum of all subjects, as well as healthy volunteers, immediately after the accident, one month after the accident and six months after the accident. In addition, cortisol levels in saliva were measured five times a day at the three above-mentioned time points to determine the circadian rhythm. In general, children with PTSD had significantly higher levels of catecholamines and significantly higher levels of cortisol in their saliva in the evening and afternoon one month after the accident than those who did not develop PTSD and the control group. After 6 months, the cortisol level normalised, while the noradrenaline level continued to increase, which may explain the relationship between stress hormone levels in adults with chronic PTSD (normal or low cortisol level) [128,129]. This could potentially explain the latency in the occurrence of PTSD after a traumatic event.

## 3. Emotion Dysregulation

Emotion dysregulation arises from disruptions in large neuronal loops involving the amygdala, insula, hippocampus, ACC and PFC [130]. In neurofunctional imaging, people with PTSD show an excessive amygdala response to negative stimuli (e.g., trauma recall), which manifests itself in negative emotionality [130,131]. Greater activity to negative faces and trauma-evoking images is also recorded in the insula. Importantly, increased activity in this area is maintained even after patients are asked to relax, which may indicate an inability to separate from traumatic memories, not only on an experiential level, but also on a neuroanatomical level [132]. The ACC, which integrates incoming information and determines the degree of amygdala involvement, is less active in people with PTSD. Reduced activity can occur in the ventral part (vACC), resulting in altered emotional judgement, or in the dorsal part (dACC), leading to problems with emotional conflict resolution, or in both parts simultaneously [130]. The simultaneous increased activation of the amygdala and hippocampus may promote better recall of traumatic events and their better retrieval in the future. At the same time, the increased engagement of the hippocampus in encoding trauma-specific images may be associated with a decreased ability to accurately recall the content [133]. The most important consequence of increased hippocampal activity is the over-generalisation of their personal response to negative stimuli [130]. Regarding the PFC, PTSD patients show a reduced involvement of the medial parts (dorsomedial prefrontal cortex, DMPFC, and ventromedial prefrontal cortex, VMPFC), which are activated in response to emotional stimuli. The DMPFC is responsible for self-regulation of emotions, while the VMPFC integrates information from subcortical structures involved in emotion processing. Reduced activity of the medial prefrontal lobes is associated with increased activity of the amygdala [131].

People with PTSD show higher availability and greater stability of mGluR5 in the PFC compared to healthy controls [134]. This promotes contextual fear conditioning after stress, fear memory generalization and correlates with the severity of avoidance symptoms [134,135,136]. Avoidance, in turn, is considered a factor that exacerbates the course of PTSD and impairs functioning [137,138]. It has been suggested that mGluR5 dysregulation in the orbitofrontal cortex may explain the higher prevalence of impulsive behaviours (i.e., self-harm, aggression and alcohol abuse) as well as suicidal ideation in individuals with PTSD, indicating a role for glutamatergic excitation in emotional dysregulation [134,139,140].

There are genetic factors involved in emotion dysregulation [141]. Serotonin transporter linked polymorphic region (5-HTTLPR) polymorphisms can contribute to affect dysregulation because they influence the magnitude and duration of serotonergic neurotransmission and are also responsible for 10% of the variance in amygdala activity [142]. There are three variants of the 5-HTTLPR allele (the short (S) allele, the long rs25531(G) (La) allele, and the long rs25531(A) (La) allele) [143]. The S allele reduces the effectiveness of transcription of the *5-HTT* gene promoter and 5-HT uptake in lymphoblasts, and at the clinical level, it is responsible for 7–9% of the hereditary variance of anxiety-related personality [144]. Individuals with the S allele and long rs25531(G) (Lg) have reduced serotonin transporter mRNA transcription, which correlates with the severity of emotion dysregulation. They also show prolonged cortisol activity after stressor exposure. In addition, individuals with the S allele have an increased risk of developing a disorganised attachment style [141].

Another genetic correlate of emotion dysregulation is the polymorphism of ankyrin repeat and kinase domains of the D2 receptor gene (*DRD2*). Children with the Taq1 allele showed greater sensitivity and emotionality to negative feedback and also tended to downplay their own successes [145]. In children, the SNP (T allele in rs4675690) near the *CREB* (cAMP response element-binding protein) gene can change the response to negative stimuli, correlating with greater activity in the anterior dorsal cingulate gyrus, the right putamen, the right caudate nucleus and the left anterior temporal pole in a state of sadness. This can increase the risk of emotion dysregulation in adulthood [146].

Emotion dysregulation is significantly associated with the chronic course of PTSD, regardless of comorbid factors such as depression, exposure to interpersonal trauma or the presence of PTSD symptoms at the time of the trauma. This means that emotion dysregulation is a traceable risk factor that can be evaluated to select the most vulnerable individuals [147]. Identifying individuals with high levels of emotion dysregulation at the time of trauma and implementing treatments designed to improve emotion regulation could aid in decreasing the development of chronic PTSD among these at-risk individuals.

Table 2 summarizes the most important information about the molecular correlates of emotion dysregulation.

## 4. Dissociation

Dissociation is a common part of the clinical picture of PTSD and is considered one of the strategies for surviving traumatic experiences from which there is no escape [148,149,150,151]. Its manifestations can include “negative” symptoms such as depersonalization, derealization, emotional numbing, analgesia, immobility as well as “positive symptoms” (intrusions) [44,150,152]. In CPTSD a more complex structural dissociation occurs [45,47]. Due to a different clinical and biological picture, a dissociative subtype of PTSD is sometimes distinguished [153,154,155,156]. Compared to the non-dissociative subtype, people with severe dissociation show a different pattern of neuronal activity at the level of cortical and subcortical structures involved in emotion processing and cognitive processes. In particular, they show enhanced activity in the PFC areas involved in emotion regulation and inhibition of the limbic system [157]. In the study by Hopper (2007) et al., which used responses to script-driven imagery scale (RSDI), dissociation primarily correlated positively with activation of the left mPFC and negatively with activity of the right insular cortex. The authors associate this with passive mental disengagement or detachment from emotional processing [158]. Importantly, the responses are different when processing conscious fear and unconscious fear. As Felmingham et al. showed, during processing of unconscious fear, people with dissociation have significantly increased activation in both amygdalae, while the non-dissociative type is characterised by an increased response in the right rostral ACC (rACC). When processing conscious fear, the dissociative group showed significantly lower activity in the right dorsomedial superior frontal gyrus, left middle frontal gyrus, right medial frontal gyrus and right inferior frontal gyrus, as well as a significantly greater response in the left ventral ACC. In addition, people with high dissociation had significantly higher activity in some subcortical structures (left pallidum, both amygdalae, both insular cortices and the left thalamus). They showed markedly high activation in the ventral PFC that is responsible for regulating emotions [159]. This shows that patients with dissociative PTSD have a different neural profile, with a predominant response from the ventral PFC in conscious processing and a response from the amygdala in non-conscious processing [157,158,159,160]. As discussed above, increased activation of the amygdala is associated with increased arousal, which, combined with the fact that it is a reaction to non-conscious fear, gives some insight into experiences and difficulties of people with dissociative PTSD. Dissociation has a negative impact on functioning [161,162,163], correlates with severity of symptoms [164,165] and suicidality [161], but it does not determine worse therapy outcomes and reduces the chances of successful therapy [166,167].

Although the occurrence of dissociation is inherently related to exposure to a traumatic experience, a biological predisposition is also postulated. Genetic factors that have been linked to dissociation are the SNPs in *FKBP5*, *SLC6A4* and *COMT*, described above. The GWAS analysis conducted by Wolf et al. also indicated the SNP rs263232 in the adenylate cyclase 8 (*ADCY8*) gene [168]. This gene codes for a Ca^2+^/calmodulin-sensitive isoform of adenylate cyclase 8 (AC8), which catalyses the conversion of ATP into cAMP. cAMP is important for synaptic plasticity, memory formation and the regulation of the HPA axis [168,169,170,171]. Although the SNP did not meet the GWAS significance criterion (i.e., *p* < 5 × 10^−8^), it is worth noting and addressing in further research. Among individuals lacking the risk allele, 17% had positive scores on Clinician-Administered PTSD Scale (CAPS) items related to dissociation, compared to 34% of individuals with one or two copies of the risk allele. AC8 deficiency can prevent or impair the consolidation of information and decoding of memories, thus causing dissociative reactions. Animals with AC8 deficiency were insensitive to risk, context and experience, and also had a dysregulated HPA axis [168,171]. The SNPs in *K1AA1456* and *KAT2B* also seem to be related to dissociation. Basing on the report that the density of *K1AA1456* in the dorsolateral PFC is lower in schizophrenia [172], Wolf et al. suggest that *K1AA1456* may contribute to the dysregulation of epigenetic processes in the PTSD dissociative subtype, which seems to be an interesting concept that requires further research. *KAT2B* has not been associated with mental disorders so far [168].

Emotion dysregulation may mediate the relationship between PTSD symptoms and dissociation. In particular, two dimensions of emotion regulation—alexithymia and the inability to use emotion regulation strategies—are predictors of dissociation [173]. One possible approach to this problem is abnormal stimulus discrimination [174], which seems to follow logically from the hyperarousal and attention bias discussed above.

Table 3 presents the most important information about the molecular correlates of dissociation.

## 5. Re-Experiencing

Re-experiencing is considered an example of pathological over-engagement at the neurobiological level [158] with involuntary and uncontrollable sensory impressions and the sense of “nowness” [175] and embodied components of self-experience [176,177]. In imaging studies, the severity of re-experiencing has been correlated with the activity of the right insular cortex. This area is responsible for somatic aspects of emotional states, including acute stimulation of the sympathetic nervous system. At the same time, a negative correlation with the activity of the left rACC has been demonstrated, which may indicate emotion dysregulation [158]. Under physiological conditions, rACC inhibits the reactivity of the amygdala. Similar correlations have been achieved in other studies with regard to the diminished mPFC activity during recollection of stressful events in individuals with PTSD [177].

Changes in the glutamatergic system also appear to play an important role in the development of re-experiencing. Increased glutamate levels found in individuals with PTSD correlate with decreased levels of the hippocampal neuronal marker N-acetylaspartate, resulting in an increased Glu/NAA ratio. Rosso et al. demonstrated a correlation between the Glu/NAA ratio in the right hippocampus and re-experiencing [106]. The hippocampus was studied because re-experiencing is thought to be one of the manifestations of hippocampal atrophy caused by glutamate excitotoxicity. Glutamate damages hippocampal neurons, leading to deficits in memory and associative learning, which is one of the underlying causes of re-experiencing [106,178,179]. The search for genetic correlates of re-experiencing is a very current topic among researchers. This symptom is associated with facilitated conduction in the cAMP pathway. In mice studies, it has been suggested that upregulation of cAMP signalling transduction enhances the retrieval and maintenance of fear memories. Transcriptome analysis in mice and humans showed that increased severity of re-experiencing symptoms occurs in people and mice with reduced mRNA expression of phosphodiesterase 4B (*PDE4B*). This is an enzyme that breaks down cAMP. Reduced mRNA expression correlated positively with reduced methylation of the corresponding locus. Research by Hori et al. shows that facilitated signalling of the cAMP pathway (with PDE4B downregulation) enhances traumatic memories [180]. In turn, GWAS studies conducted on more than 160,000 veterans identified eight regions that may play a role in re-experiencing, three of which had *p* < 5 × 10^−10^. These were *CAMKV*, *CRHR1* and *TCF4*. *CRHR1* has already been described as a gene involved in steroid signalling and the stress response. Another important locus identified is *HSD17B11*, which encodes hydroxysteroid 17-beta dehydrogenase 11, another enzyme in the steroid metabolism pathway. The connection of the *TCF4* gene, which encodes transcription factor 4, and *MAD1L1* (MAD1 Mitotic Arrest Deficient Like 1) with re-experiencing was also demonstrated, which is interesting because until now these loci were associated with schizophrenia [181,182] and schizophrenia and bipolar disorder [181,183], respectively. This indicates a certain similarity between re-experiencing and hallucinations. This hypothesis seems to be supported by the effectiveness of risperidone in individuals with re-experiencing [184]. However, it is worth noting that auditory hallucinations are not reserved to schizophrenia, as they may be present in individuals with post-traumatic, affective, personality, dissociative and eating disorders alike [185,186]. Gelernter et al. also point to the role of calcium signalling, e.g., at *CAMKV* (CaM Kinase Like Vesicle Associated) loci. These results were statistically significant only for one part of the sample (European American soldiers, EAs), while no significant associations were found in the African American (AAs) group [181].

Table 4 presents the most important information about the molecular correlates of re-experiencing.

## 6. Biology—Environment Interplay

Since the late 1990s, there has been extensive research in the search for genetic correlates of PTSD. A number of studies have noted a higher prevalence of PTSD in monozygotic twins than in dizygotic twins, indicating that there is a genetic component to the disorder [25,187,188]. It has also been noted that PTSD is more common in families of people diagnosed with PTSD, e.g., children of Holocaust survivors are more likely to have PTSD [189,190,191,192]. Interestingly, a stronger predictor than a parent’s diagnosis of PTSD itself was the parent’s exposure to traumatising events [192]. This was a landmark study on the transgenerational nature of trauma, but it did not allow for direct inference regarding the genetic component of the disorder. Indeed, a separation of genetic susceptibility from the influence of shared environmental conditions proves to be a major methodological problem.

In the study by Stein et al., it was shown that the magnitude of environmental factors is the same among women and men, although environmental factors per se remain gender specific. It also appears that the magnitude of the genetic component is different depending on the type of trauma (assaultive vs non-assaultive) [193]. Assaultive trauma develops as a result of being intentionally harmed by another person, e.g., military combat, rape, kidnapping, captivity, torture, being shot at, being stabbed, sexual violence other than rape, robbery, being threatened with a weapon and physical assault. Non-assaultive trauma is in turn a result of events that do not involve intentional harm from another person. These include for example natural disasters, car accidents, witnessing a traumatic event, learning about traumatic events experienced by a loved one or sudden death of a loved one [194,195]. Non-assaultive trauma was mainly dependent on environmental factors, while assaultive trauma could best be explained by the interaction of genetic and environmental factors [188]. Such results were achieved in both combat stress and assaultive trauma studies. The best explanatory model for non-assaultive trauma was shared environment (e.g., family) (39%) and unique environment (61%). For assaultive trauma, genetic factors appear to significantly influence the likelihood of being exposed to trauma. This is explained by the fact that genetic factors condition the way an individual responds to environmental factors [196].

This strong GxE covariance seems fully consistent with the complexity of pathogenesis and symptomatology of PTSD. In the case of CPTSD, the importance of environmental factors gains even greater significance, as the specificity of this disorder is chronic exposure to traumatic events from which there is no escape. These events often originate in childhood, when the individual is practically deprived of the opportunity to independently choose their environment, and their emotionality, personality and attachment style are formed.

Pervanidou et al. emphasise the impact of early childhood trauma on neuroendocrine regulation. Psychological trauma in childhood, adolescence or even foetal life can affect the developing central nervous system, including the areas involved in stress reactions (the PFC, hippocampus and amygdala). The risk of increased neuronal loss and delayed myelination has been demonstrated in animal models [197]. Repeated exposure to traumatic triggers causes dysregulation of the sympathetic nervous system and the HPA axis, which predisposes to mental and somatic disorders (e.g., type 2 diabetes, atherosclerosis, osteoporosis, immune system disorders, obesity, schizophrenia, anxiety disorders, depression and borderline personality disorder) [198,199,200,201,202]. The typical neuroendocrine profile of children who have experienced a traumatic event is hypoactivation of the HPA axis (low cortisol) and hyperactivation of the noradrenergic system (increase in circulating catecholamine levels), which is distinctive for PTSD [85].

The serotonin transporter gene (*5-HTT* and *SLC6A4*) seems to be an important risk factor for the development of PTSD and, at the same time, a clear example of GxE interaction. In this context, the critical part of the gene is the *5-HTT* linked polymorphism region (5-HTTLPR) located in the promoter region. Having the short variant of the 5-HTTLPR is linked to greater sensitivity to stress stimuli and, under unfavourable environmental conditions, a predisposition to the development of certain mental disorders, including depression [203,204]. The mediating role of 5-HTTLPR between environment and genotype is emphasised, especially since the gene is subject to significant epigenetic modifications [205,206]. Individuals with two risk factors (S allele and adverse prenatal or early childhood events) have significantly lower serotonin transporter mRNA expression than individuals without risk factors. Such molecular changes constitute a vulnerability factor for the development of PTSD in the event of adverse environmental factors. In the case of childhood abuse, this risk was 56.3% higher [207]. The S/S polymorphism is much more common in patients with PTSD [208]. Although the unfavourable 5-HTTLPR genotype (one or two S alleles) does not in itself determine the occurrence of PTSD, it increases the risk of developing the disorder when combined with traumatic experiences in childhood and/or adulthood. The risk is highest in people who have experienced both early childhood and adult trauma and have the 5-HTTLPR polymorphism [209]. Similarly, Stein et al. have shown that individuals with S alleles and higher exposure to maltreatment have significantly higher anxiety sensitivity, which is understood as a predisposition to develop anxiety disorders, including PTSD, and depression [210].

An unfavourable GxE interaction can promote impaired functioning from early childhood. Fox et al. conducted a longitudinal study in which the social functioning of children aged 14 months and 84 months was assessed in relation to social support perceived by the mother. Children with the S allele and low social support showed social inhibition and shyness at the age of 7 [211]. Even more important than social support seems to be the responsiveness of the mother, which translates into the development of attachment style. In children with a low genetic risk (LL alleles), there is no significant relationship between maternal responsiveness at 7 months and attachment style. In contrast, children with at least one S allele have a high risk of developing insecure attachment if they are raised by unresponsive mothers [212]. Longitudinal studies on distress intolerance in adolescents have shown that individuals with two S alleles have a lower stress resilience, and that emotional abuse in childhood is a moderator of this relationship [213]. The interplay between genetic susceptibility and the environment is consistent with changes at the neuronal and endocrine level. Homozygous S/S individuals with a history of stressful life events (SLEs) show increased arousal in the right amygdala and increased cortisol secretion in response to fearful faces. Alexander et al. detected increased functional coupling between the right amygdala and the hypothalamus, which may represent a link between neuronal and endocrine hyperactivity in S’S’/high SLEs [214].

## 7. Conclusions

Disorders associated with stress seem to be very distinct from other mental disorders. Their prominence has been recognised in the ICD-11, with the distinction of CPTSD being an innovation within category 06 (mental, developmental and behavioural disorders). As we have attempted to show, PTSD is a widely researched topic but also a huge scientific challenge due to its very complex psychopathology, which is sometimes difficult to distinguish from the effects of traumatisation at earlier stages of life. This is particularly problematic in the case of early childhood trauma and complex trauma, as it is hardly possible to separate the influence of the environment from innate susceptibility. Nevertheless, knowledge about the molecular and environmental characteristics of PTSD and CPTSD offers hope for more effective treatment of this problem. Advances in genetic diagnostic techniques can potentially facilitate screening those at risk of developing PTSD (e.g., descendants of trauma survivors or victims of childhood abuse). Analysis of neuroendocrine parameters of the HPA axis and catecholaminergic system can not only serve as a component of PTSD risk assessment but also lay the groundwork for interventions to prevent other common diseases (e.g., cardiovascular diseases). Considering the high prevalence of PTSD and CPTSD and their personal and social burden, there is a need for continuous research that can improve patients’ quality of life.

Trauma-focused interventions such as trauma-focused cognitive-behavioural therapy (TF-CBT), prolonged exposure therapy and eye movement desensitization and reprocessing (EMDR) are the most evidence-based therapeutic methods [215,216]. Of these, TF-CBT and EMDR lead to the greatest reduction in CPTSD symptoms in veterans, refugees and victims of domestic violence [217]. It is worth emphasising that these methods treat PTSD as a biologically based disorder [218,219,220], which suggests that understanding the molecular basis of these disorders seems essential for organising an effective diagnostic process and therapeutic interventions.

The molecular approach is also crucial in terms of developing effective pharmacotherapy options for PTSD and CPTSD. The medications used include selective serotonin reuptake inhibitors, serotonin-norepinephrine reuptake inhibitors, atypical antidepressants (mirtazapine, trazodone and nefazodone), alpha-adrenoreceptor antagonists (prazosin), atypical antipsychotics (risperidone and quetiapine), benzodiazepines and mood stabilising medications (lamotrigine, tiagabine and topiramate) [67,221,222]. Of these, only sertraline and paroxetine are registered by the Food and Drug Administration for PTSD [222,223], and no medication has high quality evidence [224]. Monotherapy with paroxetine, sertraline and venlafaxine has moderate recommendation. Also, no augmentation or combination has a strong evidence base at this time [224].

The variety of symptoms in each patient requires an individual approach. Attempts to find an effective combination of drugs can be a long process and can often prove ineffective. The potential new therapeutic targets include the molecular anomalies discussed in this article [225]. Ketamine, as an NMDA receptor antagonist, is attracting considerable attention as a potential new drug. Several studies have indicated that ketamine may be a rapid and effective pharmacological intervention for PTSD [226,227,228,229,230]. One of its potential advantages is supporting the extinction of original trauma memories and thus the reduction in re-experiencing [231]. However, the current state of knowledge does not allow ketamine to be introduced as a common drug for PTSD. Extensive longitudinal studies are needed to assess the usefulness of ketamine in the pharmacotherapy of PTSD [232,233]. Attention should be drawn to potential undesirable effects of ketamine on specific PTSD symptoms; for example, it has been suggested that ketamine may promote dissociation [234]. Good results were also observed in adjunctive treatment with lamotrigine (especially in terms of self-harm and aggression) [235,236,237,238] and with memantine [239,240]. Lamotrigine is a sodium channel blocker that stabilizes cell membranes and reduces presynaptic glutamate release [241], and memantine is an uncompetitive NMDA antagonist [242]. These findings seem to confirm the potential of drugs targeting the glutamatergic system in the pharmacotherapy of PTSD.

The main limitation of this review is heterogeneity of the analysed studies in terms of methodology, especially sample selection. Researchers used different inclusion and randomization criteria, e.g., structured interviews according to different protocols vs. different self-report questionnaires. Moreover, people with different types of trauma (e.g., early childhood trauma, war trauma, sexual assault and accident), age at the time of the traumatic event, and duration of the disorder took part in studies, which may distort the understanding of molecular processes in the course of PTSD. The multitude of factors that can cause PTSD makes it very difficult to draw universal conclusions about the biological basis of this disorder.

At the same time, it should be recognized that we are constantly exposed to the risk of personal psychological injuries as well as mass events that can lead to PTSD. Examples of the latter are the 9/11 attacks, the Utøya massacre, wars in Ukraine and the Middle East, the COVID-19 pandemic, or the earthquakes in South-East Asia. Researchers and clinicians should strive to deepen their understanding of PTSD both at the biological and psychological levels, as few mental disorders are as complex as this one in terms of biology–environment interactions.

## Figures and Tables

**Table 1:** Summary—neurobiological correlates of hyperarousal in PTSD and their consequences.

Mechanisms	Consequences
**‘Innate alarm system’ hyperactivation** ↑ activation of cerebellar–limbic–thalamo–cortical network	↑ visual scanning ↓ attention regulation ↓ response inhibition
**Left-to-right mPFC shift** ↓ left mPFC activity ↑ right mPFC activity ↓ corpus callosum volume	↑ stress reactivity ↑ emotional analysis ↓ cognitive analysis ↓ inhibition of HPA axis
**HPA axis hypoactivity** ↑ number of GR receptors in pituitary gland pituitary hypersensitivity to cortisol ↓ cortisol	prolonged and ↑ arousal to threat
***FKBP5* polymorphisms** ↓ sensitivity of GR to cortisol	
**GABA/Glu imbalance** ↑ Glu, ↓ GABA hippocampal damage pathological neuroplasticity in amygdala and PFC	↑ neuronal excitation neurotoxicity hyperactivation of amygdala
**Adrenergic hypersensitivity** ↓ alpha-2 autoreceptors ↑ noradrenaline secretion	↑ physiological stress response
***COMT* polymorphism (Val158Met)** ↓ COMT activity ↓ hippocampal activation	↓ resilience to stress problems in extinguishing fear memory impairment

**Table 2:** Summary—neurobiological correlates of emotion dysregulation in PTSD and their consequences.

Mechanisms	Consequences
**↑ amygdala activity**	negative emotionality excessive response to negative stimuli
**↑ hippocampal activity**	inaccurate recollection of memories over-generalization of response to negative stimuli
**↑ insular response to negative stimuli**	inability to separate oneself from traumatic memories
**↓ ACC activity**	altered emotional judgement (vACC) problems with emotional conflict resolution (dACC)
**↓ mPFC activity**	impaired emotional self-regulation impaired information integration
**↑ mGluR5 availability and stability**	fear generalization ↑ avoidance impulsive behaviours
***5-HTTLPR* polymorphism** ↓ 5-HTT mRNA transcription ↓ 5-HT reuptake in lymphoblasts	problems with extinguishing stress reactions ↓ affect regulation ↑ risk of disorganized attachment style
***DRD2* polymorphisms** ↑ dorsal cingulate gyrus activity ↑ right putamen activity ↑ right caudate nucleus activity ↑ left anterior temporal pole activity	↑ sensitivity to negative stimuli underestimating one’s achievements

**Table 3:** Summary—selected neurobiological correlates of dissociation in PTSD and their consequences.

Mechanisms	Consequences
**↑ left mPFC activity** **↓ right insular cortex activity**	detachment from emotional processing
**↑ amygdalae activity in unconscious fear**	↑ arousal
**↑ left vPFC activity in conscious fear**	emotional detachment
***ACDCY8* polymorphism** AC8 deficiency	impairment of memory consolidation HPA axis dysregulation

**Table 4:** Summary—selected neurobiological correlates of re-experiencing in PTSD and their consequences.

Mechanisms	Consequences
**↑ right insular cortex activity**	↑ somatic symptoms of emotional stress
**↓ left rACC activity**	emotional dysregulation ↓ inhibition of amygdala
**↑ Glu/NAA in right hippocampus**	inaccurate recollection of memories over-generalization of responses to negative stimuli
**↑ insular response to negative stimuli**	inability to separate from traumatic memories
**↓ *PDE4B* expression** ↑ cAMP signalling transduction	↑ retrieval of traumatic memories

## Data Availability

Not applicable.
